# Bone pathologic fracture revealing an unusual association: coexistence of Langerhans cell histiocytosis with Rosai-Dorfman disease

**DOI:** 10.1186/s12907-017-0044-1

**Published:** 2017-04-07

**Authors:** Boubacar Efared, Asmae Mazti, Badarou Chaibou, Gabrielle Atsame-Ebang, Ibrahim Sory Sidibé, Layla Tahiri, Fatimazahra Erregad, Nawal Hammas, Abdelmajid El Mrini, Hinde El Fatemi, Laila Chbani

**Affiliations:** 1 Departement of pathology, Hassan II University hospital, Fès, Morocco; 2 Department of orthopaedics and traumatology, Hassan II University hospital, Fès, Morocco; 3 Faculty of Medicine and Pharmacology, Sidi Mohamed Ben Abdellah University, Fès, Morocco

**Keywords:** Langerhans cell histiocytosis, Rosai-Dorfman disease, Pathology

## Abstract

**Background:**

The coexistence of Rosai-Dorfman disease (RDD) with Langerhans cell histiocytosis (LCH) is very rare, as to date only 17 cases have been reported in the english literature. The pathophysiology of this uncommon co-occurrence still remains enigmatic and a subject of various speculations.

**Case presentation:**

We report a case of a 30-year-old female patient who presented with a pathologic fracture of the left proximal femur. Her medical history was unremarkable, there were no fever, skin lesions, lymphadenopathy or other organomegaly at physical examination. X-ray radiograph of the fractured femur showed an isolated and ill-defined osteolytic lesion. The histopathological analysis of biopsies from this lesion were consistent with a combined RDD-LCH of the bone.

**Conclusion:**

Combined RDD-LCH is a very rare phenomenon, whose pathophysiology still remains unclear and a subject of various speculations.

## Background

Histiocytic disorders are a are and heterogenous entity comprising a large variety of diseases with a wide spectrum of clinical, histological, molecular and prognostic features [1, 2]. Langerhans cell histiocytosis (LCH) and Rosai-Dorfman disease (RDD) are among the most common component of the large group of histiocytic disorders [1, 3].

Recent studies suggested that LCH is a clonal proliferation of abnormal dendricytic cells (Langerhans cells) with a particular phenotype, CD1a+, S-100+ and CD68+ [3]. The disease can affect one organ or involve several organs or systems, such as skin, bones, lymph nodes, the nervous system, the spleen or the liver [1, 4].

In contrast, RDD is a non-neoplastic disease characterized by a polyclonal proliferation and a tissue accumulation of CD1a- histiocytes [2, 3, 5]. Like LCH, the disease can involve one organ, especially lymph nodes, but extra-nodal sites account for approximately 25–43% of all cases [2, 6]. Most common extranodal sites include the skin, nasal cavity, soft tissue, bones, salivary glands and central nervous system [2].

However, the co-occurrence of RDD and LCH is a very rare phenomenon. Since the first case of combined RDD-LCH reported in 2002 by Wang KH et al. [7], to the best of our knowledge, only 16 additionnal cases have been reported in the english literature. We report herein, another additionnal case of this rare association with the particular synchronous occurrence in the bone tissue.

## Case presentation

**Fig. 1:**
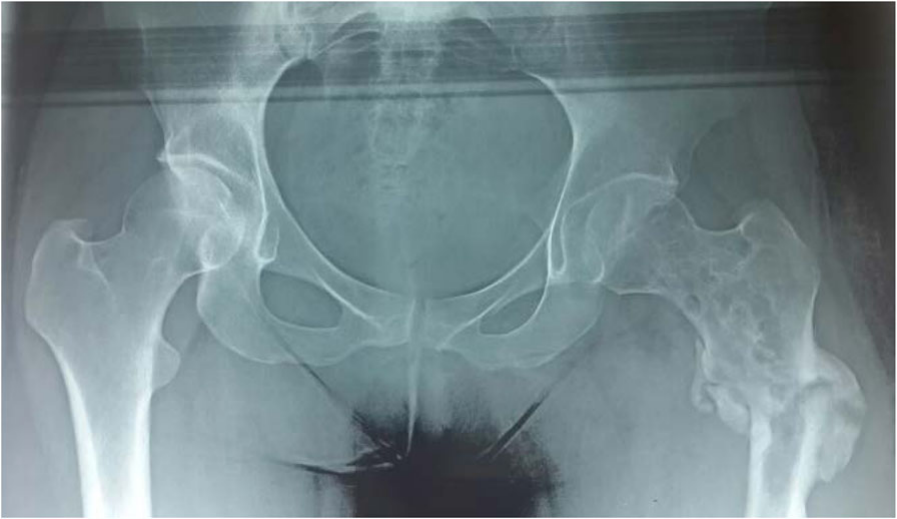
X-rays of the hip showing an osteolytic tumor at the site of the fracture (*left side*)

A 30-year-old woman was admitted at emergency department for a pathologic fracture of the left femur bone, after a mild trauma. The patient’s medical history, as well as her family history were unremarkable. The physical examination showed a painful and swollen left hip with inability to move the lower limb because of the pain. Apart from these trauma-related signs, there were no fever, skin lesions, lymphadenopathy or other organomegaly. An X-ray radiograph has been performed and revealed at the traumatic site, a relatively well-limited trochanteric osteolityc lesions at the fracture’s site (Fig. 1). No other lesions have been discovered. The biological check-up was within normal limits. Biopsies have been performed for histopathological evaluation.

**Fig. 2:**
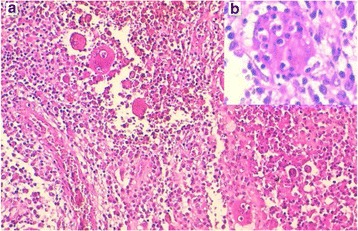
A dense infiltrate composed of numerous plasma cells, lymphocytes, neutrophils, eosinophils, macrophages and prominent figures of emperipolesis (**a**). Higher magnification showing a figure of emperipolesis. A huge cell with abundant pink cytoplasm, engulfing plasma cells, lymphocyts and neutrophils is seen (**b**)

**Fig. 3:**
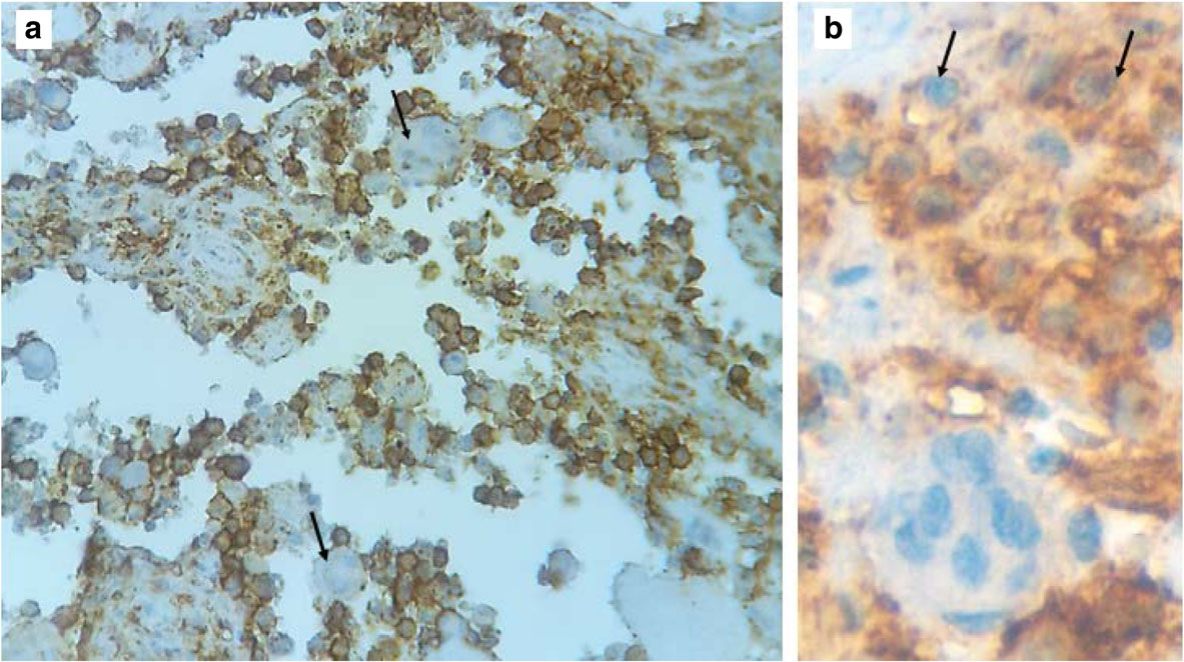
Mononuclear cells stained strongly positive for CD1a, whereas giant cells with emperipolesis stained negative (*black arrows*) (**a**). Some CD1a + cells are round-shaped with oval nuclei, reminiscent to RDD-cells (“Blanks cells” or transitional cells) (**b**)

**Fig. 4:**
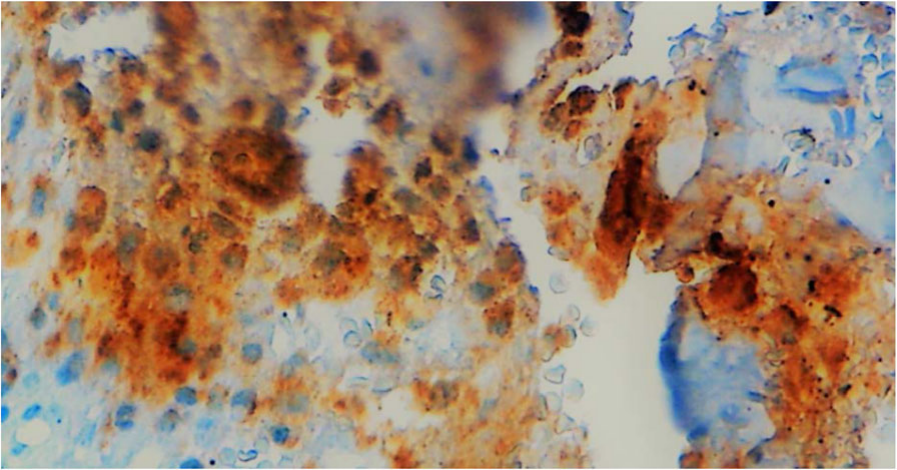
S-100 protein is strongly expressed by histiocytes of both RDD and LCH components

After treatment with 10% nitric acid (decalcification), the histological examination of the submitted bone biopsies showed a dense inflammatory infiltrate destroying the bone tissue (Fig. 2a). It consisted of admixed numerous plasma cells, lymphocytes, neutrophils, eosinophils and histiocytes. Prominent emperipolesis figures are seen with large histiocytes that engulf inflammatory cells such as plasma cells, neutrophils and macrophages (Fig. 2b). Beside these emperipolesis figures, there are some large histiocytes with oval nuclei and granular chromatine and inconspicuous nucleoli. Also, in some areas, scattered cells with “bean-shaped” and grooved nuclei are visible. At imunohistochemistry, many mononucleated cells show intense membraneous expression of CD1a, whereas giant multinucleated cells with emperipolesis stain negative (Fig. 3a). Also, some CD1a positive cells are round-shaped with oval nuclei, and a large cytoplasm, morphologically reminiscent of RDD cells (Fig. 3b). These cells have overlapping features between RDD and LCH cells, they have been called “transitional cells” [4]. However, both cell types express CD68 and S-100 protein (Fig. 4). Finally, the diagnosis of combined RDD and LCH has been made. The patient recovered well after surgery (osteosynthesis and curetage) without any sign of the disease.

## Discussion

The association between RDD and LCH is extremely rare, since the first case reported by Wang KH et al.[7], 16 other additional cases have been reported in the english literature [4, 5, 7–13] (Table 1) through single-reported cases, except for the 9 reported cases (largest series) by O’Malley DP et al. [4] and one case among a series of 25 cutaneous RDD in China, reported by Kong YY et al. [8]. To the best of our knowledge, to date we have a total of 18 reported cases of association between RDD and LCH (RDD-LCH). Thus, the average age is 29.55 years (15 months–68 years), with a female sex predilection, 6 males for 12 females (Sex ratio = 1 M/2 F). The pediatric population is about one third of all cases (6 cases under the age of 18). In most cases, at initial presentation, the disease is limited to one organ (a total of 15 cases) : 9 cases in lymph nodes, 5 in the skin, 1 case in the bone (our current case) [4, 7–11, 13]. In fact, our patient is the only case among all reported cases to have a synchronous association of RDD-LCH in the bone. The remaining cases had a double-organ disease : 1 case reported by O’Malley et al. had subcutaneous and abdominal disease [4], Cohen-Barak E. et al. described a case with initial occipital bone LCH, that secondarily presented with skin RDD one month later after treatment [5]. Similarly, the case reported by Kutty SA et al. had initial cranial bone LCH, and presented 2 years later with a RDD in the dura mater [12]. Also, among cases reported by O’Malley DP et al. 2 had a particular presentation : one case presented initially with lymph node LCH, with recurrence as RDD 10 months later, the second case had combined RDD-LCH with a recurrent RDD after 4 years [4]. In sum, all reported recurrences showed only RDD ; the pattern of association is either synchronous, RDD-LCH in one organ (like our case), or a metachronous combination as initial LCH with/without RDD, that presents secondarily as recurrent RDD in the same organ or another different organ [4, 5, 12].

**Table 1:** Reported cases of combined RDD-LCH

Authors	Year	No. of cases	Age (years)	Sex	Site
Wang KH et al. [7]	2002	1	45	F	Skin
Kong YY et al. [8]	2007	1	52	F	Skin
Sachdev R et al. [9]	2008	1	3	M	LN
O’Malley DP et al. [4]	2010	9	25^a^	2 M/7 F	LN : 8 Abdominal mass/SC : 1
Llamas-Velasco M et al. [10]	2012	1	68	M	Skin
Cohen-Barak. E et al. [5]	2014	1	10	M	Skin-Bone
Jin W et al. [11]	2014	1	20	F	Skin
Kutty SA et al. [12]	2015	1	31	M	Bone-dura mater
Litzner BR et al. [13]	2015	1	48	F	Skin
Our case	2016	1	30	F	Bone

^a^the average age of the 9 reported cases; *F* female sex, *M* male sex, *LN* lymph node, *SC* subcutaneous

The morphologic diagnosis often shows typical features of both types of histiocytosis, RDD and LCH [4, 5]. The cells of the LCH component have the classic bean-shaped or coffee-like appearance and grooved or folded nuclei with pink and granular cytoplasm [4, 9]. These cells show no emperipolesis unlike in RDD. Variable amount of inflammatory cells, especially eosinophils are associated with LCH [4]. The immunophenotype is typically CD1a+, CD207+, CD68+, S-100+ [1, 4, 5, 9]. In areas of RDD, the most eye-catching feature is emperipolesis that shows cells with a huge cytoplasm containing intact inflammatory cells like plasma cells, lymphocytes, neutrophils, eosinophils or macrophages [2, 4, 5]. The so-called Rosai-Dorfman cells (RD-cells) have characteristic cytologic features. They are moderate to large in size with abundant, ill-defined and granular cytoplasm; nuclei are round or oval with smooth contours, vesicular and marginated chromatin with unconspicuous round nucleoli [2, 4]. RD-cells are usually surrounded by numerous inflammatory cells, such as lymphocytes, plasma cells, neutrophils and less often eosinophils [2, 4]. At immunochemistry, RD-cells express CD68, CD163, S-100, without expression of CD207 or CD1a [4]. Cells with emperipolesis have typically RD-cells immunophenotype, especially negative for CD1a and langerin (CD207). When combined in the same organ, the RDD component seems to be larger than the LCH component, that presents often as scattered CD1a + cells [4, 7, 13]. This habitual pattern is not seen at immunohistochemistry in our case. In fact, at HES-staining (Hematein-Safran-Eosin), emperipolesis was the first eye-catching appearance in our case (Fig. 2), and typical LCH cells were very rare, seen as scattered cells among the diffuse infiltrate and the accompaning emperipolesis figures (Fig. 2). But, at immunohistochemistry, most mononuclear cells were strongly CD1a positive, only some mononuclear cells and cells with emperipolesis figures were negative for this marker (Fig. 3a). A careful look at CD1a + cells shows that they have mostly a round to oval shape, and often correspond to cells that have RDD appearance at HES-staining (Fig. 3b). These aspects were strongly suggestive of what O’Malley DP et al. called for the first time in their largest series of 9 cases of RDD-LCH, “Banks cells”, an eponym related to one of the co-authors (Peter M Banks) that initially brought these cells to other authors attention [4]. These “Banks cells” were a minor component of lesions (less than 5% of all lesional cells), which had morphologic features comparable to RDD, with larger round nuclei and open chromatin and increased cytoplasm, but with CD1a and Langerin expression. These cells seemed to display transitional features between a lesional cell of LCH and RDD [4]. So, in accordance with these authors, we could say that our current case is a “Banks cells”-riched case of RDD-LCH, or transitional cells-riched case.

Anyway, transitional cells present or not, the main enigmatic and challenging issue is the exact pathological and molecular mechanism underlining the association between RDD and LCH. In fact, previous studies have supported a similar cytokine-mediated mechanisms in both RDD and LCH, and it has been speculated that the RDD presents as a reaction to LCH, or that both LCH and RDD could result from divergent differenciation from a common precursor, or that the histiocytes have undergone a phenotypic switch [1, 4, 5]. LCH is considered as a monoclonal and neoplastic disease as a number of studies showed that, it is usually associated with *BRAF* mutation [1, 3]. In contrast, RDD is still supposed to be a reactive disease [1, 5]. However, O’Malley DP et al. and Cohen-Barak et al., reported some cytogenetic abnormalities associated to some combined RDD-LCH cases [4, 5]. What is obvious, in some reported RDD-LCH cases, is that the RDD appeared secondarily after initial LCH-RDD [4] or LCH [5, 12], suggesting that the RDD was a reactive response to LCH. Similar co-existence with juvenile xanthogranuloma (JXG) or secondary “transformation” of LCH to JXG, have been reported [14, 15]. JXG is another subtype of the wide spectrum of histiocytic disorders, its association with LCH, as reported previously [14, 15], was another yet enigmatic issue. Chemotherapy has been evoked as a cause of the secondary apparition of RDD after treatment of the initial LCH [5].

As suggested by previous authors, the co-existence of RDD and LCH is more than a simple coincidence, and more studies in the future are required to highlight the complex relationship between the two entities.

## Conclusion

Association between RDD and LCH is a very rare occurrence; a few cases have been reported in the current literature. The mechanism by which these entities occur together still remains a subject of various speculations, and future studies are required to unveil this enigmatic and challenging fact.

## Abbreviations

HES: Hematoxyline-eosine-safran
JXG: Juvenile xanthogranuloma
LCH: Langerhans cell histiocytosis
RDD: Rosai-Dorfman disease
